# The effects of prolonged wear of textured shoe insoles on gait, foot sensation and proprioception in people with multiple sclerosis: study protocol for a randomised controlled trial

**DOI:** 10.1186/s13063-016-1337-x

**Published:** 2016-04-21

**Authors:** Anna L. Hatton, John Dixon, Keith Rome, Sandra G. Brauer, Katrina Williams, Graham Kerr

**Affiliations:** School of Health and Rehabilitation Sciences, Therapies Building (84A), The University of Queensland, Brisbane, QLD 4072 Australia; Health and Social Care Institute, Teesside University, Middlesbrough, UK; Health and Rehabilitation Research Institute & School of Podiatry, AUT, Auckland, New Zealand; Institute of Health and Biomedical Innovation, QUT, Brisbane, Australia

**Keywords:** Gait, Shoe insoles, Foot sensation, Proprioception, Multiple sclerosis

## Abstract

**Background:**

Many people with multiple sclerosis experience problems with walking, which can make daily activities difficult and often leads to falls. Foot sensation plays an important role in keeping the body balanced whilst walking; however, people with multiple sclerosis often have poor sensation on the soles of their feet. Wearing a specially designed shoe insole, which enhances plantar sensory information, could help people with multiple sclerosis to walk better. This study will explore whether long-term wear of a textured insole can improve walking in people with multiple sclerosis.

**Methods:**

A prospective randomised controlled trial with two parallel groups will be conducted aiming to recruit 176 people with multiple sclerosis living in the community (Brisbane, Australia). Adults with a clinical diagnosis of multiple sclerosis, Disease Steps score 1–4, who are ambulant over 100 m and who meet specific inclusion criteria will be recruited. Participants will be randomised to a smooth control insole (n = 88) or textured insole (n = 88) group. The allocated insole will be worn for 12-weeks within participants’ own footwear, with self-report wear diaries and falls calendars being completed over this period. Blinded assessors will conduct two baseline assessments and one post-intervention assessment. Gait tasks will be completed barefoot, wearing standardised footwear only, and wearing standardised footwear with smooth and textured insoles. The primary outcome measure will be mediolateral base of support when walking over even and uneven surfaces. Secondary measures include spatiotemporal gait parameters (stride length, stride time variability, double-limb support time, velocity), gait kinematics (hip, knee, and ankle joint angles, toe clearance, trunk inclination, arm swing, mediolateral pelvis/head displacement), foot sensation (light touch-pressure, vibration, two-point discrimination) and proprioception (ankle joint position sense). Group allocation will be concealed and all analyses will be based on an intention-to-treat principle.

**Discussion:**

This study will explore the effects of wearing textured insoles over 12-weeks on gait, foot sensation and proprioception in people with multiple sclerosis. The study has the potential to identify a new, evidence-based footwear intervention which has the capacity to enhance mobility and independent living in people with multiple sclerosis.

**Trial registration:**

Australian New Zealand Clinical Trials Registry ACTRN12615000421538. Registered 4 May 2015.

## Background

Falls are a major threat to the health and well-being of people with multiple sclerosis (pwMS) [1, 2]. Up to 50 % of pwMS report falling within the past 6 months, and 50 % of these falls result in injuries [3]. Impaired mobility and balance are two major risk factors for falls in pwMS [2]. In one study, 85 % of pwMS reported gait disturbances as their main complaint [4] and continued loss of mobility amongst their greatest concerns for the future [5]. Impaired walking in pwMS is typically characterised by an increased mediolateral (ML) base of support, reduced stride length, step length and velocity, and prolonged double-limb support time during level ground walking, relative to healthy individuals [6–8]. Incipient signs of deteriorating walking ability can even be observed in the early stages of the disease [6–8]. Therefore, interventions that effectively preserve or enhance walking capacity are paramount to improving quality of life and maintaining independence.

Current rehabilitation strategies to improve gait and balance in pwMS predominantly involve exercise participation to address deficient motor function, with some consideration given to sensory training [9–13]. These multimodal approaches have been shown to significantly improve several clinical and functional measures in pwMS, including dynamic balance, rate of falls, physical activity levels, perceived balance confidence, walking ability and quality of life [9–13]. However, there is an urgent need to develop additional methods to complement exercise and which target MS sensory impairments [14–19] to a greater extent, in particular tactile sensation and proprioception, in order to preserve and enhance mobility for as long as possible. Previous evidence has shown that a strong relationship exists between foot sensation and standing balance performance in pwMS [15]. Similarly, a loss of lower limb proprioception, including joint position sense at the ankles and feet, in pwMS can detrimentally affect gait and standing balance, leading to greater dependence on compensatory motor mechanisms in order to remain upright [17, 19]. An increasing body of literature suggests footwear interventions may be another treatment option to help improve gait performance in pwMS [20–22].

Textured shoe insoles, designed to enhance plantar sensory information, have been shown to consistently alter gait patterns in the short-term, potentially improving walking stability in a range of clinical populations, including older fallers [23], adults with Parkinson’s disease [24] and pwMS [20, 21]. To date, exploratory studies indicate that textured insoles can lead to beneficial alterations in spatiotemporal gait parameters such as a reduced ML base of support [20], improved gait kinetics and kinematics [21] in pwMS. Significant increases in lower limb muscle activity during both stance and swing phases of gait, changes in knee and hip excursion and ground reaction forces, have been found immediately after pwMS wore textured insoles, with these changes attributed to enhanced stimulation of plantar mechanoreceptors [21]. Furthermore, after wearing textured insoles for 2 weeks, significant increases have also been observed in stride and step length, and significant decreases in the size of the ML base of support during level-ground walking – interpreted to represent a more confident gait pattern. These changes were observed independent of wearing the textured insoles, again supporting the theory that a sensory training effect may have occurred during the intervention period [20]. However, recent evidence reports no significant changes either in spatiotemporal gait measures during treadmill walking or plantar sensitivity after wearing textured insoles over a longer, 4-week intervention period in pwMS [25]. It is possible that any effects of textured insoles on gait may only be identified when walking in conditions that emulate everyday life [25]. Further, whilst no changes were observed in plantar sensitivity, alterations may have occurred in other measures of sensory function such as foot proprioception [25]. As such, the short-term effects of textured insoles on mobility, and their proposed underlying mechanisms in pwMS, remain unclear. It is possible that the benefits of textured insoles in pwMS may accrue, and that additional benefits may be observed, with prolonged wear over 4-weeks, but this has not yet been explored. Previous work has shown limited effects of textured insoles on gait and balance measures in pwMS immediately after wearing the insoles for the first time, with subsequent improvements observed following 2-weeks of wear [20].

This randomised controlled trial will determine whether wearing textured shoe insoles for 12-weeks can improve gait when walking over even and uneven surfaces in pwMS. The primary aim of this study is to explore whether prolonged wear of textured insoles alters ML base of support (as a measure of walking stability) from baseline assessment 2 to the post-intervention assessment. Secondary aims are to explore whether prolonged wear of textured insoles alters other spatiotemporal gait parameters including stride length, stride time variability, double-limb support time and gait velocity, gait kinematics (specifically lower limb joint and trunk movement), and changes in the perception of foot sensation or proprioception, as underlying mechanisms associated with improvements in spatiotemporal gait parameters.

## Methods

### Design

**Fig. 1:**
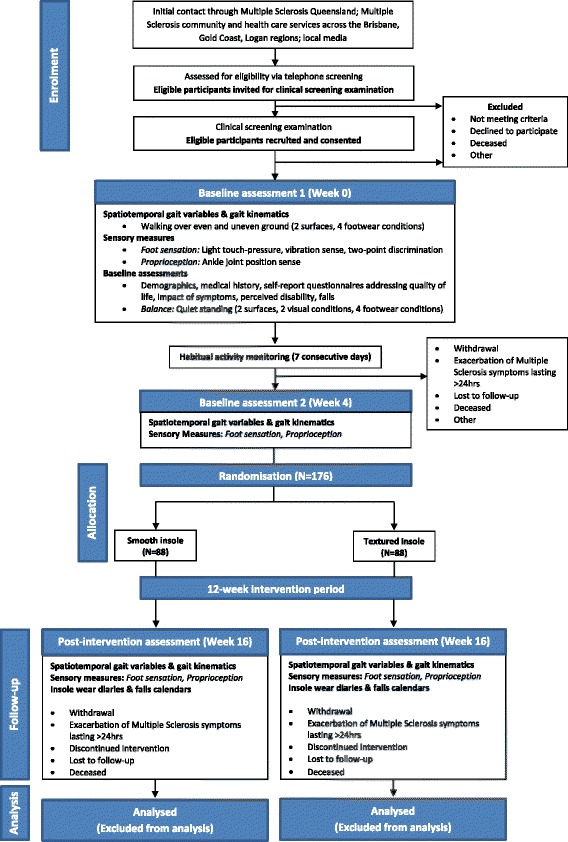
Trial design

A prospective, parallel group, single blinded, randomised controlled trial with 176 pwMS living in the community will be conducted, conforming to the Consolidated Standards of Reporting Trials guidelines [26] (Fig. 1).

### Sample size

Sample size has been calculated for the primary outcome measure, ML base of support during even surface walking, based on our pilot data [20]. Our preliminary study reported mean (SD) readings at baseline for base of support of 13.78 (5.11) cm and a significant mean change of –1.66 cm (*P* = 0.02) at 2-weeks post intervention. With a power of 80 %, and alpha level of 0.05, a calculation for two related groups indicated that 76 participants were required in each group. In our pilot study, we recruited 46 pwMS, with no loss to follow-up across two visits (although completion of all test procedures was limited by fatigue in some participants). As this randomised controlled trial involves a longer intervention period, we will allow for a 15 % attrition rate. An 85 % retention rate over a 16-week period (baseline assessments at week 0 and week 4, intervention 12-weeks, post-intervention assessment at week 16) is appropriate based on previous MS intervention studies. Three randomised controlled trials with 12-week intervention periods conducted in pwMS, report retention rates of 82 % [27], 88 % [11], and 90 % [28]. Therefore, 88 participants per group will be recruited, giving a total of 176 participants.

### Location and setting

All assessments will be conducted in the Gait Laboratory within the Institute of Health and Biomedical Innovation at Queensland University of Technology, Brisbane, Australia.

### Participants

Men and women with a diagnosis of MS will be identified through a pool of sampling frames including MS Queensland, local MS health care providers and community organisations across the Brisbane, Gold Coast and Logan regions, Australia. Participants will be recruited through mainstream media advertisements and written materials distributed to individuals listed on the MS Queensland database and those attending local MS clinics. Recruitment procedures will be centrally coordinated by clinical staff working within each organisation to maintain patient confidentiality. Participants will be invited to voluntarily contact the Principal Investigator for further information.

Participants will be eligible to take part if they meet the following criteria: aged over 18 years; clinical diagnosis of MS; ambulant over 100 metres with or without the use of an assistive device; and Disease Step rating of 1–4 [29]. Participants rated as Disease Step 1 (mild disability: mild symptoms and/or signs) to 4 (late cane: unable to walk 25 feet without a cane/unilateral support) will be eligible to take part in this study, assuming they have sufficient ambulatory capacity to complete the gait trials.

Exclusion criteria are neurological conditions other than MS; peripheral neuropathy; currently being prescribed over-the-counter or custom-made foot orthoses; cardiovascular or orthopaedic conditions including recent injury to the back or legs limiting ambulation; unstable psychiatric condition or cognitive impairment (Short Form Mini-Mental State Examination score <24) [30]. Furthermore, enrolled participants who report an exacerbation of MS symptoms persisting >24 h, 4 weeks prior to, or at any time during, the intervention period will also be excluded from the study. All participants will initially be screened via telephone interview and invited to attend a clinical examination to confirm eligibility. Written informed consent will be obtained from all participants. This study was approved by the Medical Research Ethics Committee at The University of Queensland (#2014000781) and University Human Research Ethics Committee at Queensland University of Technology (#1500000615).

### Randomisation and blinding

The concealed randomisation schedule will be established using a computer-generated random number sequence, and maintained by an offsite investigator who is neither involved with the enrolment nor with assessment of participants. Consecutively numbered, randomly ordered, opaque envelopes containing group allocation (in a 1:1 ratio), will be opened consecutively after baseline assessment 2, by a second research assistant who is only responsible for administering the insoles. All investigators and the first research assistant, who are involved in the enrolment or assessment of participants over the duration of the trial, will remain blinded to group allocation. Following baseline assessment 2, the Principal Investigator and first research assistant will leave the gait laboratory to ensure blinding to the insole condition. The second research assistant will then fit the participant with their allocated insole, and provide advice regarding the frequency of wear, completion of insole wear diaries, and emergency contact details for local podiatry care. Participants will be instructed not to divulge their group allocation. As it is not possible for participants to be blinded to their allocated group (those in the intervention group will be able to perceive the textured material against the sole of their foot), the full aims of the study will be concealed. Participants will not be told that the intervention is designed to provide enhanced plantar sensory information which could potentially lead to changes in gait. Such knowledge could influence how participants walk and they could purposefully alter their walking patterns between conditions; debriefing will occur upon completion of the study. Furthermore, coding of participants will not refer to group.

### Intervention

In this randomised controlled trial we will investigate two different shoe insoles: textured insoles and smooth (control) insoles. Both insoles have been implemented in previous research strategies in pwMS [20], older fallers [23] and middle-aged adults [31]. The textured insole (Evalite Pyramid ethyl vinyl acetate (EVA), 3 mm thickness, shore value A50, black, OG1549; Algeos PTY Ltd., Liverpool, UK) was selected from a range of EVA soling materials, and has small, pyramidal peaks with centre-to-centre distances of approximately 2.5 mm. The smooth control insole (Medium Density EVA, 3 mm thickness, shore value A50, black, OG1304; Algeos PTY Ltd., Liverpool, UK) was chosen from a range of plain EVA materials and has a flat surface with no indentations. Insoles will be tailored to each participant’s shoe size. An experienced podiatrist will oversee and advise on the delivery of insoles, and any podiatry-related issues including insole fit, durability and dermatological or peripheral changes at the foot during the intervention period. Participants will be instructed to wear their allocated insoles, in their own shoes, as much as possible. All assessments of balance and gait will be conducted with the participants wearing standardised footwear (Donated by Pacific Brands Australia Pty Ltd), comprising a basic construct rubber-soled shankless shoe with a soft canvas upper [32], into which the insoles will be inserted. This standardisation will control for any possible insole/shoe interactions across participants, which could impact the findings. To allow for familiarisation to the footwear, participants will be instructed to walk for 5 minutes in the standardised shoes prior to testing.

### Primary outcome measures

#### Spatiotemporal gait variables

The primary gait measure will be ML base of support when walking over an even and uneven surface. Our pilot study demonstrated that after 2-weeks wear of the textured insoles, the significant mean reduction in base of support was 1.7 cm (*P* = 0.02) compared to baseline measures [20]. The magnitude of this effect is highly clinically relevant as previous research indicates a mean difference of ~2 cm in base of support exists between pwMS and healthy controls [6, 7]. This suggests that the textured effect is clinically significant, and may be of sufficient magnitude to reduce base of support to a level similar to healthy adults.

### Secondary outcome measures

#### Spatiotemporal gait variables

Additional measures of walking stability will include stride length, stride time variability, double-limb support time, and gait velocity, when walking over an even and uneven surface. Our pilot study reported that wearing textured insoles for 2-weeks led to significant increases in mean stride length (right leg: 5.8 cm (*P* < 0.01); left leg: 4.4 cm (*P* < 0.01)), compared to baseline assessment [20]. Details of specific methods underpinning all measures are provided in the assessment section below.

#### Gait kinematics

During both even and uneven surface walking trials, lower limb gait kinematics will be collected using a 3D motion capture system and will include hip, knee and ankle joint angles (and their inter-relationships) and foot-to-floor angle to determine maximum toe clearance. Segmental measures of trunk inclination, as well as arm swing, mediolateral pelvis and head displacement will also be collected. Specific details are presented below.

#### Sensory measures

Light touch-pressure sensation will be determined by recording the smallest monofilament that the participant can perceive at five locations on the foot as detailed below [15]. Vibration sense will be measured using a digital stop watch, started when the tuning fork touches the participant’s skin at two sites on the feet, then stopped when the participant indicates the vibration can no longer be felt. The average of three trials will be recorded for both feet (seconds) [15]. For two-point discrimination, when the participant perceives two stimuli as one, the distance will be recorded in millimetres [15]. Ankle joint position sense will be determined by the participant performing the ankle joint position sense test [33].

#### Insole wear and falls

Participants will be followed for 12-weeks with insole wear self-reported diaries and falls calendars to determine (1) the number of hours insoles are worn and (2) the frequency, time, and location of any falls and injuries. In this study, a fall will be defined as an unexpected event in which the participant comes to rest on the ground, floor or lower level [34].

### Clinical screening examination

Prior to enrolment, all individuals will undergo a clinical screening examination, conducted by a Specialist Neurological Physiotherapist (KW), which will include the assessment of disease stage and symptoms including spasticity and ataxia. Stage of disease will be determined using Disease Steps [29]. This tool is an assessment of disability in patients with MS, which has low inter-rater variability, correlates strongly to the Expanded Disability Severity Scale at initial assessment (EDSS), and can be used to monitor disease progression [35]. Spasticity will be assessed using the Tardieu Scale [36], and ataxia scored using the Brief Ataxia Rating Scale [37].

### Baseline assessments

Demographics, including sex, age, height, and body mass, will be collected. To characterise the study sample, participants will be asked to complete questionnaires that address relevant medical history and medications, length of time since diagnosis of MS, current MS symptoms using the MS Impact Scale (MSIS-29) [38], and perceived walking ability using the MS Walking Scale (MSWS-12) [39]. Quality of life, the impact of fatigue and pain, and perceived disability will be assessed using four self-report questionnaires: MS Quality of Life Instrument (MS QoL-54) [40]; Modified Fatigue Impact Scale (a questionnaire which measures how MS-related fatigue affects everyday life including physical, cognitive and psychosocial functioning [41]); Medical Outcomes Study (MOS) Pain Effects Scale (a MS-specific questionnaire which assesses how pain and disturbing sensations, such as burning or tingling, affect everyday life [42]); and the Perceived Deficits Questionnaire (a MS-specific questionnaire which assesses several domains of cognitive function that are commonly affected by MS, namely attention, retrospective memory, prospective memory, planning and organisation [43]). Number of self-reported falls experienced in the previous 12 months will be recorded, and current fear of falling assessed using the Falls Efficacy Scale-International [44].

Following the clinical screening examination, all participants will complete initial assessments of gait, foot sensation and proprioception (baseline assessment 1). Standing balance and activity levels will also be measured at baseline assessment 1 only. Each participant will receive a wireless activity monitor (activPAL, Glasgow, Scotland), to be worn every day for seven consecutive days, allowing us to characterise the activity of the study group, monitor habitual weekly activity levels and establish any relationships with gait performance at baseline. The increasing use of accelerometry in pwMS [45, 46] is accredited to its ability to allow monitoring of changes in walking impairments with disease progression (e.g. worsening of MS) or disease activity (e.g. acute relapse), over long periods of time [47]. Four weeks after baseline assessment 1, a second baseline assessment (baseline assessment 2) will be conducted. The purpose of this 4-week waiting period is to establish each participant’s natural rate of MS disease progression, specifically the magnitude of change in the primary and secondary outcome measures of gait, foot sensation and proprioception, prior to delivery of the intervention.

### Gait

Gait performance will be evaluated by completing a 12 m walk over an even surface and an uneven surface. The even surface will consist of a level, vinyl material: the top cover of an instrumented walkway (GAITRite^®^, CIR Systems, Inc., Havertown, PA 19083, USA). The GAITRite^®^ system is an electronic walkway, approximately 8.2 m long (the active area being 0.61 m wide and 7.32 m long), which has been shown to have high reliability [48, 49]. The uneven surface (placed directly on the laboratory floor, adjacent to the GAITRite^®^ walkway) will consist of two layers of thick soft foam, over which small blocks of wood of uneven shapes and sizes will be spread in a random manner, with a top layer of artificial grass covering the walkway, using previously described methods [50]. Maintenance of stability when walking requires individuals to control their centre of mass within a constantly changing base of support; this becomes even more challenging when the surface is uneven, increasing the risk of loss of balance, resulting in a fall. Deficits in balance control during walking or, conversely, the therapeutic benefit of interventions (such as shoe insoles) on walking performance may only become apparent when the balance challenge is sufficiently demanding. The uneven walking surface will emulate a situation encountered in daily life. A start and finish line will be marked on the floor 2 m in front and 2 m behind both the even and uneven surface walkways, allowing participants to accelerate and decelerate outside the walkways [48]. Participants will be positioned at the start line and instructed to walk at their comfortable, self-selected walking pace. Five walking trials will be completed on the even surface and five trials on the uneven surface, each whilst barefoot, wearing standardised footwear only, and wearing two different shoe insoles (textured and smooth) within standardised footwear. The test sequence (footwear condition, surface) will be randomised. Spatiotemporal gait variables will be measured using the GAITRite^®^ system (sampling rate 80 Hz) when walking over the even surface, and using an 11-camera Vicon^®^ motion capture system (Vicon, 6 × MX13 and 5 × T40 cameras, giganet control box, with a MX Net and Mx Link), sampled at 200 Hz, when walking over the uneven surface. Participants will have multiple reflective markers attached to their body, following the Vicon PlugIn Gait full body model. The Vicon system records the position of reflective markers placed at standardised anatomical sites on the upper and lower body and will be used to measure spatiotemporal gait variables and gait kinematics.

### Balance

Standing balance will be assessed to provide a measure of basic, unperturbed postural stability. Participants will stand on an AMTI force platform (sampling rate 1000 Hz), using a standardised foot position (heels placed 1/10th participants height apart and angled to 14° [51]), and arms hanging by their sides, for 30 seconds [52]. Double-limb standing tests will be performed on a firm and foam surface, with their eyes open and eyes closed. To prevent vestibular disruption when standing with eyes open, participants will be instructed to look straight ahead and focus on the middle of a black circular visual target (10 cm diameter), mounted onto a board positioned 3 m from the centre of the force platform, and adjusted to the eye level of each participant. Standing balance will be assessed whilst barefoot, wearing standardised footwear only, and when wearing two different shoe insoles (textured and smooth) within standardised footwear. The test sequence (footwear condition, surface, vision) will be randomly presented. Measures of baseline standing balance will include centre of pressure path velocity, range and standard deviation of centre of pressure movement in the anterior-posterior and ML directions.

### Foot sensation and proprioception

Somatosensory function, including light touch-pressure sensation, vibration sense, and two-point discrimination, will be assessed. Semmes-Weinstein monofilaments (smallest (1.65) to largest (6.65)) will be used to determine light touch-pressure sensation at five locations on the foot, namely the plantar surface of the great toe, first metatarsal head, fifth metatarsal head, heel, and dorsum of the foot between the first and second toes [53]. The monofilaments will be applied perpendicular to the skin for 1.5 seconds, and the participant will be required to indicate whether the fibre can be felt. The smallest monofilaments (1.65–4.08) will be applied three times consecutively, whilst larger ones (4.17–6.65) will be applied only once [15]. Duration of vibration sense will be measured using a 128-Hz frequency tuning fork at the first metatarsal head and medial malleoli of both feet [15]. The ability to distinguish between two light-touch stimuli (two-point discrimination) will be measured using an aesthesiometer applied to the skin at three foot regions: tip of the great toe, first to second metatarsal interspace, and fifth metatarsal head. Each region will be touched with either one or two points simultaneously in a random order, with approximately 2 seconds between each application of the stimuli. Assessment will begin with the two stimuli at the maximum distance apart, and decrease until the participant can no longer differentiate the two points [15]. Foot position awareness will be assessed bilaterally using the ankle joint angle reproduction test [33]. The investigator will passively set the participant’s ankle joint to three pre-determined angles in plantarflexion and dorsiflexion directions, relative to a neutral foot position. A variable time and trajectory will be used when positioning the foot in order to eliminate extraneous cues and psychophysical processes. The participant will be asked to reposition the ankle joint at the target angle by moving only the foot segment. Accuracy in joint positioning will be determined by measuring the difference between the target and actual angles using an internet-based goniometer [54]. This application has been shown to be a valid method for measuring joint angles and has a high level of inter-rater (ICC2,1 = 0.96 to > 0.99) and intra-rater (ICC = all > 0.99) reliability [54].

### Post-intervention assessment

Gait, foot sensation and proprioception will be assessed within 2 weeks of the end of the 12-week intervention period, using the same procedures employed at baseline. A 12-week intervention period will provide maximal time to allow for the accrual of any sensory training effects and accumulation of meaningful changes in outcome measures, in particular for participants with MS who show minimal gait disturbance at baseline and currently engage in an active lifestyle. This intervention period is consistent with previous randomised controlled trial intervention studies conducted in pwMS [11, 27, 28], and footwear intervention trials [55, 56]. This final point of assessment will (1) quantify whether any immediate changes in gait, observed at baseline, have accrued over time, or if additional effects can be seen and (2) determine whether there are any alterations in the perception of foot sensation or proprioception, which may suggest the insoles have a sensory training effect. Participants will be asked to return their insole wear diaries and falls calendars at this time. Participants will also be asked to rate the level of comfort experienced when wearing the insoles by way of a series of 100 mm visual analogue scales used in previously published research [57].

### Data analysis

All analyses will be conducted in a blinded manner, on an intention-to-treat basis, with the alpha set to 0.05. We will explore frequency distributions, percentages and calculate means and standard deviations for the outcome measures. Differences between intervention and control groups in spatiotemporal gait variables, gait kinematics, foot sensation or proprioception, over the intervention period, will be explored using General Linear Models (repeated measures analysis of variance, ANCOVA), in a two group (smooth control insole; textured insole) by three phase (baseline assessment 1, baseline assessment 2, post-intervention) model. We will adjust for potential confounding variables (e.g. age, sex, disease duration) by using these as covariates. Non-parametric tests will be used where data is not normally distributed or violates the assumption of sphericity. Multiple regression modelling will be used to determine any relationships between foot sensation, proprioception and measures of gait performance. Data will be analysed using SPSS version 22 (SPSS Inc., Chicago, IL 60606, USA).

## Discussion

Gait impairment is one of the most disabling and debilitating complaints reported by pwMS [5]. Deteriorating mobility observed in the early stages of disease [6–8] not only increases the risk of falling [1, 2], but frequently culminates in a complete loss of walking ability in the advanced stages [58]. The associated personal and societal burdens can have devastating implications for the individual, their families and national health services. Physical rehabilitation strategies reported to improve gait in pwMS commonly involve short-term multi-component exercise programs [9–13]. Maintenance of walking stability is attributed to optimal sensorimotor function; however, therapeutic management of gait impairments in pwMS largely focuses on addressing motor problems and poor aerobic capacity, and to a lesser extent sensory training, which is commonly addressed purely by way of balance tasks under a variety of sensory conditions. Interventions targeting sensory impairments at a more local level, including foot sensation and lower limb proprioception, are not frequently incorporated. This is a crucial area to address, as loss of foot sensation and impaired lower limb proprioception are strongly associated with standing balance and gait performance in pwMS [15, 19]. Therefore, the effectiveness of current strategies for managing mobility in pwMS could be further enhanced by using a wider range of treatment techniques.

Providing enhanced sensory input to the plantar surface of the feet has recently been considered a potential mechanism through which footwear interventions may improve gait [21, 22, 24, 59–63] by way of altering sensorimotor function. Underlying physiological mechanisms by which a textured insole may initiate changes in gait are suggested to include the provision of sufficient tactile stimulation to alter the rate of discharge from mechanoreceptors or firing patterns of populations of sensory afferents located in the feet. Textured shoe insoles appear to have the capacity to alter gait patterns, potentially improving gait stability in ageing, neurodegenerative and neuromuscular disease groups with known balance impairments. To date, exploratory studies report that wearing shoe insoles deigned to enhance plantar sensation can significantly increase single-limb support time [24], increase stride length and reduce double-limb support time [32] during walking in people with Parkinson’s disease. Similar conclusions are emerging for pwMS, with exploratory work observing beneficial alterations in spatiotemporal gait parameters [20], gait kinetics and kinematics [21].

This randomised controlled trial will use fundamental knowledge of sensory and motor function in MS to develop novel ways to improve gait by way of enhancing sensory information at the soles of the feet. Preliminary work in this clinical population [20] provides strong evidence of improvements in gait patterns when textured insoles were worn (as a single intervention) for 2 weeks. It is possible that the benefits of wearing textured insoles may accrue, and that additional benefits may be observed, over a longer period of time. Findings from this trial could have implications on the management of gait impairment in pwMS. The benefit for pwMS (and their families) is that this study may lead to the development of a new, evidence-based footwear intervention which is inexpensive, non-invasive, promotes self-management by the user, and has the capacity to enhance mobility and independent living. Furthermore, addressing problems with mobility, and subsequently quality of life, could have a major economic impact through improvements in productivity or reducing working days lost. The benefit for healthcare professionals is that this study may generate vital evidence to inform the development of more effective, multi-faceted and multi-disciplinary rehabilitation programmes, which are tailored to address a greater range of MS-specific impairments that contribute to deteriorating gait. This could have major implications on current clinical guidelines and policy relating to physical rehabilitation strategies for pwMS.

### Trial status

Ongoing.

## Abbreviations

EVA: Ethyl vinyl acetate
ML: Mediolateral
MS: Multiple sclerosis
pwMS: People with multiple sclerosis
